# Metabolism of Zearalenone and Its Major Modified Forms in Pigs

**DOI:** 10.3390/toxins9020056

**Published:** 2017-02-08

**Authors:** Sabina B. Binder, Heidi E. Schwartz-Zimmermann, Elisabeth Varga, Gerlinde Bichl, Herbert Michlmayr, Gerhard Adam, Franz Berthiller

**Affiliations:** 1 Christian Doppler Laboratory for Mycotoxin Metabolism and Center for Analytical Chemistry, Department of Agrobiotechnology (IFA-Tulln), University of Natural Resources and Life Sciences, Vienna (BOKU), Konrad-Lorenz-Str. 20, 3430 Tulln, Austria; sabina.binder@boku.ac.at (S.B.B.); elisabeth.varga@boku.ac.at (E.V.); franz.berthiller@boku.ac.at (F.B.); 2 BIOMIN Research Center, Technopark 1, 3430 Tulln, Austria; gerlinde.bichl@biomin.net; 3 Department of Applied Genetics and Cell Biology, University of Natural Resources and Life Sciences, Vienna (BOKU), Konrad-Lorenz-Str. 24, 3430 Tulln, Austria; herbert.michlmayr@boku.ac.at (H.M.); gerhard.adam@boku.ac.at (G.A.)

**Keywords:** mycotoxins, masked mycotoxins, plant metabolites, bioavailability, cleavage, enzymatic hydrolysis, gastrointestinal tract, high performance liquid chromatography tandem mass spectrometry

## Abstract

The *Fusarium* mycotoxin zearalenone (ZEN) can be conjugated with polar molecules, like sugars or sulfates, by plants and fungi. To date, the fate of these modified forms of ZEN has not yet been elucidated in animals. In order to investigate whether ZEN conjugates contribute to the total ZEN exposure of an individual, ZEN (10 µg/kg b.w.) and equimolar amounts of two of its plant metabolites (ZEN-14-*O*-*β*-glucoside, ZEN-16-*O*-*β*-glucoside) and of one fungal metabolite (ZEN-14-sulfate) were orally administered to four pigs as a single bolus using a repeated measures design. The concentrations of ZEN, its modified forms and its mammalian metabolites ZEN-14-glucuronide, α-zearalenol (α-ZEL) and α-ZEL-14-glucuronide in excreta were analyzed by high-performance liquid chromatography tandem mass spectrometry (HPLC-MS/MS) based methods. The biological recovery of ZEN in urine was 26% ± 10%, the total biological recovery in excreta was 40% ± 8%. Intact ZEN-14-sulfate, ZEN-14-*O*-*β*-glucoside and ZEN-16-*O*-*β*-glucoside were neither detected in urine nor in feces. After ZEN-14-sulfate application, 19% ± 5% of the administered dose was recovered in urine. In feces, no ZEN metabolites were detected. The total biological recoveries of ZEN-14-*O*-*β*-glucoside and ZEN-16-*O*-*β*-glucoside in the form of their metabolites in urine were 19% ± 11% and 13% ± 7%, respectively. The total biological recoveries in urine and feces amounted to 48% ± 7% and 34 ± 3%. An explanation for the low biological recoveries could be extensive metabolization by intestinal bacteria to yet unknown metabolites. In summary, ZEN-14-sulfate, ZEN-14-*O*-*β*-glucoside, and ZEN-16-*O*-*β*-glucoside were completely hydrolyzed in the gastrointestinal tract of swine, thus contributing to the overall toxicity of ZEN.

## 1. Introduction

Mycotoxins are natural secondary metabolites produced by various species of fungi and are able to exert toxic effects in humans, animals, and plants [1]. Zearalenone (ZEN), one of the most important mycotoxins, can cause fertility and reproduction disorders in mammals. Generally, gilts are the most sensitive species to ZEN exposure [2]. ZEN possesses an estrogenic effect, which manifests itself clinically in hyperestrogenism. Prepubertal females react particularly sensitively [3]. As reviewed by Minervini and Dell’Aquila [4] the main reason for ZEN sensitivity is that swine convert ZEN to the more estrogenically active α-zearalenol (α-ZEL). Typical symptoms of ZEN toxicosis in gilts and sows are reddening, hyperemia, and edematous swelling of the vulva, enlargement of the uterus with the formation of cysts on the ovaries and enlargement of the mammary glands. In addition, it can also cause a vaginal or rectal prolapse. Edematous swelling of the preputium and the mammarian complex, as well as atrophy of the testes and reduced concentration of spermatozoa are characteristic impacts on boars [5,6].

**Figure 1:**
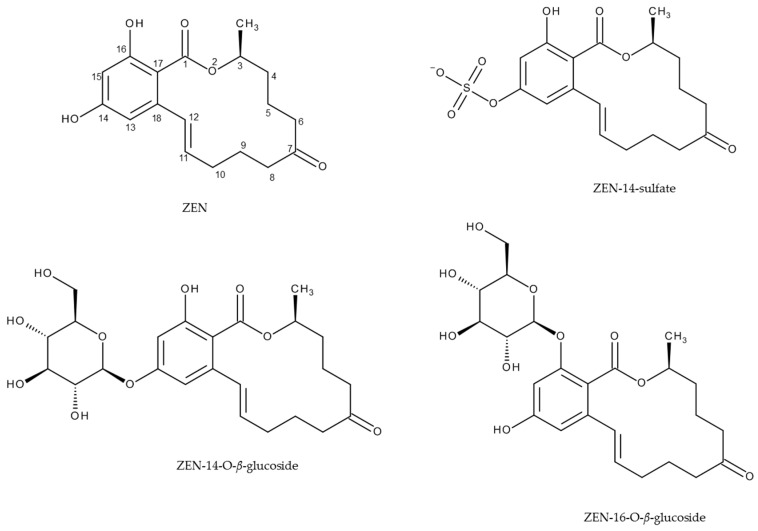
Structures of zearalenone (ZEN) and its plant and fungal metabolites.

ZEN can frequently be found in grains and cereals, in particular in wheat, corn, and products thereof [7]. In addition, plants and fungi can metabolize ZEN by attaching polar residues, like glucose or sulfate. Mycotoxin conjugates formed by plants are called masked mycotoxins [8]. Examples are zearalenone-14-*O*-*β*-glucoside (ZEN-14-Glc) and zearalenone-16-*O*-*β*-glucoside (ZEN-16-Glc) (see Figure 1). Zearalenone-14-sulfate (ZEN-14-S, see Figure 1) is an example for a ZEN conjugate of fungal origin. The sum term comprising plant and fungal mycotoxin metabolites as well as any other mycotoxin derivative formed by biological or chemical processes is “modified mycotoxins” [9]. 

Formed by *Rhizopus* sp., ZEN-14-Glc was first identified as metabolite of ZEN in 1986 [10]. The presence of ZEN-14-Glc in naturally contaminated wheat was first reported in 2002 in the course of a small scale survey on wheat grain samples [11]. In that study, the relative proportion of ZEN-14-Glc to ZEN in wheat grain was up to 30%. In a comprehensive study on the occurrence of modified mycotoxins in food and feed, ZEN-14-Glc was found in fiber-enriched bread, bran-enriched bread, breakfast cereals, oatmeal samples, and most feed samples [12]. The ratio of ZEN-14-Glc to ZEN was highly variable in food products produced in two subsequent years and ranged from 1:36 in 2010 up to 1:1.3 in 2011. 

In 2006, Poppenberger et al. [13] reported the first UDP-glucosyltransferases (UGT) gene encoding an enzyme from *Arabidopsis thaliana* which is able to convert ZEN into ZEN-14-Glc. They also showed that the interaction of ZEN-14-Glc with the human estrogen receptor was interrupted by conjugation of ZEN to glucose, suggesting reduced toxicity of ZEN-14-Glc. More recently, Kovalsky et al. [14] identified a barley UGT (*Hv*UGT14077) which can convert ZEN into the known ZEN-14-Glc and the novel masked mycotoxin ZEN-16-Glc. However, ZEN-16-Glc has not yet been detected in food or feed. 

ZEN-14-S was originally isolated from a *Fusarium graminearum* rice culture and characterized by Plasencia and Mirocha [15]. It was also formed by other *Fusarium* strains [15] and by cultures of *Rhizopus arrhizus* [16]. Like ZEN-14-Glc, ZEN-14-S is a common contaminant of cereal-, soy-, and corn-based food and feed products [12]. Low levels of ZEN-14-S were found in different commodities like whole meal wheat bread, wheat flour, bran flakes, wheat flakes, biscuits, corn meal, and cereal snack bars. In that study, ZEN-14-S to ZEN ratios were estimated to be over 1:10 [17]. In feed raw materials like soybeans, corn and wheat, maximum ZEN-14-sulfate to ZEN ratios were 1:0.5, 1:15, and 1:3, respectively [12]. 

Absorption of ZEN glucosides and sulfate in their conjugated form would likely have limited the impact on animal health, as ZEN conjugates show a drastically decreased estrogenicity at the estrogen receptor level [18]. However, the risk of consuming food or feed containing ZEN conjugates is the potential hydrolysis to their native toxic forms within the digestive tract of mammals [8]. As a consequence, the resulting parent mycotoxin could be absorbed, thus increasing the total toxin exposure of an individual.

Guidance levels for ZEN in animal feed, introduced by the European Commission, are 0.25 mg/kg in complementary and complete feeding stuffs for sows and fattening pigs, as well as 0.1 mg/kg in the same commodities for piglets and gilts [19]. The European Food Safety Authority (EFSA) Panel on Contaminants in the Food Chain (CONTAM) [20] published a no-observed-effect-level (NOEL) of 10 µg/kg body weight (b.w.) per day for pigs and established a tolerable daily intake (TDI) for humans of 0.25 µg/kg b.w. based on recent studies in pigs. In contrast to ZEN, no maximum or guidance values have been set for modified forms of ZEN, yet. In this respect, EFSA recently declared the need to set an appropriate group health-based guidance value for ZEN and its metabolites in feed and food and decided to add all ZEN metabolites in one group TDI of 0.25 µg/kg b.w. per day. Relative potency factors to ZEN for differences in in vivo estrogenicity of the different modified forms of ZEN were applied. The modified mycotoxins received the same relative potency factors as ZEN [21]. Previously, EFSA [7] also performed a risk assessment for humans and animals in order to elucidate the toxicity of modified forms of ZEN. EFSA concluded that people who regularly consume above-average amounts of cereals may exceed the TDI of ZEN 2.2 times, if the concentration of ZEN and its metabolites is closer to the upper bound level in cereals and if all ZEN metabolites are equally toxic. In order to verify that hypothesis, and to draw a conclusion on the toxicity of the compounds, it is important to investigate the cleavage of ZEN metabolites in vivo. 

Therefore, the aim of this work was to evaluate the absorption, distribution, metabolism, and excretion (ADME) of plant (ZEN-14-Glc, ZEN-16-Glc) and fungal (ZEN-14-S) ZEN metabolites in pigs for the first time. Special attention was paid to potential hydrolysis of conjugated ZEN metabolites in the gastrointestinal (GI) tract of pigs. Overall, the results of this study extend the current knowledge about the in vivo metabolization of some ZEN conjugates, thus contributing significantly to the further risk assessment of these compounds.

## 2. Results and Discussion

### 2.1. Clinical Symptoms

ZEN is able to cause different harmful health effects in pigs, not only fertility and reproduction disorders [3], but also increased oxidative stress, decreased nutrient digestibility, or reduced growth rate [22]. For animal welfare considerations, amounts of administered toxins were chosen on the basis of no-observed-effect-levels of 10 µg/kg body weight per day for oral application in pigs [20]. In short, four piglets received a single orogastric bolus of ZEN (10 µg/kg b.w.) and of its plant or fungal metabolites (at equimolar dose), respectively, using a repeated measures design. During and after the treatment no clinical symptoms were observed in any of the animals.

### 2.2. Sample Preparation and Method Performance

In order to study the metabolization of ZEN and its modified forms, two liquid chromatography-tandem mass spectrometry (LC-MS/MS) based methods for determination of ZEN and its plant, fungal and mammalian metabolites in urine and feces were developed. In addition to ZEN, ZEN-14-Glc, ZEN-16-Glc and ZEN-14-sulfate, also the mammalian metabolites α-ZEL, β-ZEL, ZEN-14-glucuronide (ZEN-14-GlcA), as well as α- and β-ZEL-14-glucuronide (α- and β-ZEL-GlcA) were included. These two methods shared a common LC-MS/MS part and differed only in the sample preparation. 

The first step in method development for analyzing ZEN and its metabolites in lyophilized feces samples was to find a suitable extraction procedure. To this end, different extraction solvents were tested by performing three subsequent extractions. Since acetonitrile (ACN)/water (50/50, *v/v*) yielded acceptable apparent recoveries for all analytes (89%–106%), and this extraction solvent was chosen for the final procedure. At an injection volume of 3 µL, the matrix effects varied between 30% for ZEN and 105% for ZEN-14-S. To minimize matrix effects, different injection volumes (1, 2, and 3 µL) were investigated. An injection volume of 2 µL provided the best compromise, achieving less severe matrix effects than the 3 µL injection volume and sufficiently low limits of detection. Signal suppression and enhancement (SSE) values between 47% for α-ZEL and 104% for ZEN-14-S and apparent recoveries ranging from 49% (α-ZEL) to 124% (ZEN-14-S) were achieved (see Table 1). The recovery of extraction (R_E_) was ≥103% for all analytes. In lyophilized feces, limits of detection (LODs) ranged from 1.8 to 22 ng/g, corresponding to 0.06 to 0.73 ng/mL in feces extracts. Limits of quantification (LOQs) in freeze-dried feces were between 5.7 and 72 ng/g, which corresponded to LOQs between 0.19 and 2.4 ng/mL in feces extracts (see Table 1).

Urine samples were diluted to the same creatinine content with water in order to obtain similar concentrations of matrix compounds in all samples [23]. Apparent recoveries varied between 84% (β-ZEL) and 113% (α-ZEL-GlcA). The repeatability of the method, expressed as relative standard deviation (RSD), was between 6% and 10%. LODs in measurement solution (urine diluted to 0.2 mM creatinine) varied between 0.02 and 0.73 ng/mL, while LOQs in measurement solution were between 0.08 and 2.4 ng/mL (see Table 2). As LODs and LOQs were determined in urine diluted to 0.2 mM creatinine, and dilution factors ranged from 3 to 143, LODs and LOQs in undiluted urine samples differed for the individual animals. Detailed results of the method validation are listed in Table 1 and Table 2.

### 2.3. Amounts of Excreted Toxins and Biological Recoveries in Urine and Feces

Piglets weighed 9.5 ± 1.3 kg at the start of the trial and their weight at the end of the trial ranged from 14.6 kg to 17.3 kg. Volumes of urine collected in the individual sampling intervals (0–24 h and 24–48 h after treatment) varied between 50 mL and 730 mL per piglet, while amounts of lyophilized feces ranged from 10 g to 197 g. Total excreted volumes and amounts of urine and feces per piglet in the two days period increased with time and were between 158 mL and 1210 mL and 29 g and 325 g, respectively. 

The bioavailability of a toxin in pigs can be assessed on the basis of the quantitative urinary recovery of the toxin [24] and its metabolites. Our results represent first in vivo data of biological recoveries of modified ZEN metabolites. The total amounts of ZEN and its metabolites excreted into urine and feces by the animals (*n* = 4) in the two time periods (0–24/24–48 h after application) are listed in Table 3. Results are expressed as nanomolar amounts to simplify comparison between the eliminated amounts of various mycotoxins. ZEN, α-ZEL, and ZEN-14-GlcA were the compounds detected at concentrations >LOD. There was a large variation in the concentrations and amounts, and also some variation in the metabolite patterns of daily excreted ZEN and ZEN-metabolites. Hence, the metabolization of ZEN and its modified forms is not only influenced by factors like animal species and route of ZEN administration, but also by variations between individuals and the status of their digestion. Biological recoveries are provided in Table 4. 

#### 2.3.1. Negative Control (Water Application)

The basal diet was analyzed before the experiment and contained approximately 3.6 ng/g ZEN. This low ZEN contamination already resulted in traces of ZEN in feces samples after oral application of water (negative control), but was considered to be of marginal impact for the further experiments. 

#### 2.3.2. Positive Control (ZEN Application)

Following oral administration of ZEN (positive control), concentrations of ZEN and ZEN-14-GlcA in urine were between <LOQ and 64 ng/mL and <LOQ and 312 ng/mL, respectively. ZEN-14-GlcA was the major ZEN metabolite in urine. In lyophilized feces, ZEN and α-ZEL were detected in concentrations up to 248 ng/g and up to 110 ng/g, respectively. β-ZEL could neither be detected in urine nor in feces samples. The total biological recovery of ZEN (sum of both sampling periods) in the form of ZEN, ZEN-14-GlcA, and α-ZEL in urine and feces was on average 40% ± 8% and ranged from 30% and 49% (see Table 4). The biological recovery of the applied toxin in urine ranged from 15% and 39%, whereas between 10% and 18% of the administered amount of ZEN was recovered in feces. 

The metabolism of ZEN in different animal species has already been investigated in several studies. In vitro studies showed that ZEN and its metabolites α- and β-ZEL are glucuronidated in animals and humans in the intestine, liver and other organs mainly at the sterically unhindered phenolic 14-hydroxy group which results in the formation of ZEN-14-GlcA [25]. In the in vitro study of Pfeiffer et al. [25], ZEN-14-GlcA and ZEN-16-GlcA were formed when ZEN was incubated with hepatic microsomes from different species in the presence of uridine 5’-diphosphate glucuronic acid (UDPGA). In all species, ZEN-14-GlcA was the predominant glucuronide, with ZEN-14-GlcA:ZEN-16-GlcA ratios greater than 9:1. Pfeiffer and et al. also showed that the hepatic microsomes of pigs had the highest activity, followed by liver microsomes of bovines, rats, and humans. Consequently, the glucuronidation rate of ZEN in pigs is likely to be faster than in other species. 

In vivo, ZEN is metabolized by reduction to α- and/or β-ZEL in the GI tract [26,27] and in the liver [28]. The ratio of α-/β-ZEL formation is species dependent [28]. In pigs, α-ZEL is the major form although variable ratios ZEN:α-ZEL:β-ZEL have been reported in the literature e.g., [29,30,31,32,33]. In a recent study with 47 pigs in the two highest dose groups, the average ratio of ZEN:α-ZEL:β-ZEL in pig urine was approximately 8:4:1 [32]. Up to now, the percentage of ZEN- and ZEL-glucuronides in urine had been estimated by measuring untreated and enzymatically hydrolyzed urine and calculating the difference of ZEN and ZEL concentrations in hydrolyzed and untreated urine e.g., [30,33]. Zöllner and et al. reported 27% glucuronidation for ZEN, 88% glucuronidation for α-ZEL and 94% glucuronidation for β-ZEL [30]. Similarly, Mirocha et al. found higher glucuronidation of ZELs than of ZEN (23% glucuronidation for ZEN, 75% for α-ZEL and 56% for β-ZEL) [33]. In general, as ZEN- and ZEL-glucuronide standards are not commercially available, the methods employed in the literature for determination of ZEN and its metabolites in pig urine, included enzymatic hydrolysis for cleavage of ZEN- and ZEL-glucuronides. In contrast to these studies, urine samples of our study were not enzymatically hydrolyzed. With that, our study is the first to quantify ZEN-glucuronide in pig urine on the basis of an authentic reference standard [34]. This approach was chosen because ZEN-glucosides potentially contained in pig urine of our experiment would at least partially be cleaved by β-glucuronidase preparations. Hence, direct quantification of ZEN-glucuronide gave the first direct estimate of the percentage of ZEN glucuronidation in pig urine. After ZEN application, glucuronidation rates between <24% and >93% were obtained in the individual pig urine samples. On average, 61% ± 32% of ZEN occurred as glucuronide. High variation in the percentage of ZEN-glucuronide in urine has previously been reported in the literature [26]. This is in contrast to nearly complete glucuronidation of ZEN in plasma and might be due to autohydrolysis of urinary ZEN-glucuronide during storage and/or analysis [26]. 

As reported by Dänicke et al. [31], the terminal elimination half-life of intravenously administered ZEN is 2.63 h. In our study, the major part of ZEN and its metabolites (20% ± 11% of the administered dose) was excreted into urine within the first 24 h after application. The second greatest part was eliminated via feces 24–48 h after application (12% ± 4%). One reason for this time difference might be extensive biliary excretion of ZEN and its metabolites. As shown by Biehl et al. and Dänicke and et al. [26,31], the terminal elimination half-life of ZEN was greatly shortened in animals whose enterohepatic cycling had been interrupted by removal of bile. In pigs with an intact enterohepatic cycling, the amount of radiolabeled [^3^H]ZEN metabolites excreted via urine was twice as high as the amount eliminated via feces. In addition, the major part of the radioactive dose in urine was excreted within the first 24 h [26]. Our results are in line with these findings. The biological recoveries of administered ZEN in our work fall within those obtained in previous studies, in which pigs were fed diets with a single or combination of mycotoxins. For example, in the study of Gambacorta et al. [24], the mean percentage of ingested ZEN excreted into urine 24 h after application was 28.4%. Another example is the study of Olsen et al. [35], in which 14%–16% of the applied ZEN was excreted as ZEN and α-ZEL in urine eight hours after administration. In contrast, Dänicke et al. estimated the biological recovery of intravenously administered ZEN to 77% [31]. However, the major part (44%) was eliminated via urine within 12 h and had, therefore, not passed the GI tract. Furthermore, as biological recoveries of orally administered radiolabeled ZEN were 67% after 48 h [26], it is very likely that extensive metabolization of ZEN by intestinal bacteria to yet unknown metabolites had taken place in the orally-dosed pigs of our study.

#### 2.3.3. Administration of ZEN-14-S

Following oral administration of ZEN-14-S, the analyte itself was neither detected in urine nor in feces samples. In urine collected 24 h after treatment, ZEN concentrations were up to 69 ng/mL. Only traces were detected in the second sampling period. ZEN-14-GlcA (81 ng/mL) and traces of α-ZEL were detected in urine of two different animals. 

In the study of Plasencia and Mirocha [15], ZEN and ZEN-14-S, respectively, were applied intragastrically to rats (*n* = 6 per treatment) and their effect on uterus weight was examined. The study showed that after ZEN-14-S ingestion in rats, it partially retained the estrogenic activities of ZEN. 

In our study, ZEN-14-S was rapidly hydrolyzed in the pigs’ GI tract. Since neither ZEN-14-S nor ZEN were found in the feces (see Table 3 and Table 4), it can be assumed that the applied amount was too low, so that the products of hydrolyzed ZEN-14-S were excreted only in urine, even after enterohepatic cycling. For instance, Biehl et al. showed that about 85% of radioactive ZEN which was excreted in bile was reabsorbed into the systemic blood system and then redistributed to tissues and subsequently eliminated in urine [26]. Yet, extensive GI metabolization to unknown metabolites cannot be excluded, either.

After oral ZEN-14-S application, the total recovery (in both sampling periods) of all metabolites in urine was between 14% and 24% with an average of 19% ± 5% (see Table 4). It should be emphasized here that lyophilized ZEN-14-S was dissolved in water directly on site to prevent cleavage before administration. Our study is the first which shows in vivo data on the fate of ZEN-14-S in pigs. 

#### 2.3.4. Administration of ZEN-14-Glc and ZEN-16-Glc

Following oral administration of ZEN-14-Glc and ZEN-16-Glc, none of the glucosides could be detected in urine or feces. In urine samples of piglets orally dosed with ZEN-14-Glc, concentrations of ZEN varied between 40 and 62 ng/mL, while the concentrations of ZEN-14-GlcA ranged from <LOQ to 95 ng/mL. α-ZEL could be found in urine of one animal at a concentration of 20 ng/mL. In feces samples of the pigs treated with ZEN-14-Glc, concentrations of ZEN and α-ZEL ranged from 17 to 198 ng/g and from 46 to 179 ng/g, respectively. After ZEN-14-Glc application, the total biological recovery (in both sampling periods) of its metabolites in feces and urine was between 40% and 56% with an average of 48% ± 7% (see Table 4). The biological recovery in feces ranged from 21% to 40%, while the biological recovery in urine was between 11% and 35%.

In urine samples of piglets fed with ZEN-16-Glc, concentrations of ZEN varied between 4.0 ng/mL and 45 ng/mL. Concentrations of 11 and 12 ng/mL of α-ZEL were determined in urine of two animals. Likewise, traces of ZEN-14-GlcA were detected in urine of one animal. In feces samples of piglets orally dosed with ZEN-16-Glc, concentrations of ZEN between 28 and 227 ng/g and levels of α-ZEL ranging from 35 to 109 ng/g were found in both sampling periods. After ZEN-16-Glc application, the total biological recovery of its metabolites in feces and urine collected up to 48 h after treatment was between 31% and 39% with an average of 34% ± 3% (see Table 4). The biological recovery of its metabolites in feces and urine ranged from 16% to 31% and from 3% to 18%, respectively.

The metabolism of several mycotoxin glucosides has been studied in recent years. Dall’Erta et al. [36] used a human in vitro model to study deoxynivalenol-3-glucoside (D3G), ZEN-14-Glc, ZEN-14-S and their parent compounds by imitating different steps of the digestive process—more precisely, the salivary, the gastric, and the duodenal step—by consecutive enzymatic treatments. The glucoside and sulfate of ZEN were completely hydrolyzed within 30 min upon incubation with human intestinal bacteria. In part, ZEN was formed (61% after 30 min), but formation of other unknown metabolites took place, too, as indicated by disappearance of ZEN to only 40% of the applied molar dose of the conjugates. In absence of microbiota, mycotoxins were stable.

Gratz et al. [37] described recent in vitro results about a) the epithelial absorption of ZEN compounds (ZEN, ZEN-14-Glc, α-ZEL, α-ZEL-14-Glc, β-ZEL, and β-ZEL-14-Glc) in human Caco-2/TC7 cells using a serum-free culture medium and b) about the activity of human gut microbiota in feces samples from five donors spiked with the same ZEN compounds. In the first experiment with Caco-2/TC7 cells, 91%–93% of the starting amount of ZEN-14-Glc, α-ZEL-14-Glc and β-ZEL-14-Glc were recovered from the apical compartment after 24 h, which initially indicates a limited bioavailability of these glucosides. From the basolateral compartment, only traces of ZEN-14-Glc and α-ZEL-14-Glc were detected after six and 24 h. In the second experiment, over 97% of ZEN-14-Glc, α-ZEL-14-Glc and β-ZEL-14-Glc were hydrolyzed after incubation with human gut microbiota for four hours. However, only 40% of the ZEN-14-Glc dose was detected as ZEN and 50% of ZEN-14-Glc was converted to unidentified metabolites. Likewise, 60% of α-ZEL-14-Glc and 30% of β-ZEL-14-Glc were found as α-ZEL and β-ZEL, which means that 40% or 70% of the dose could not be recovered in the form of identified metabolites. In a recent study, Dellafiora et al. [38] also showed that human breast cancer cells (MCF7) including fetal bovine serum are able to convert ZEN-14-Glc to ZEN, α-ZEL and β-ZEL. Most recently, Dellafiora et al. treated fetal bovine serum, bovine plasma and whole blood with ZEN-14-Glc and observed in vitro hydrolysis of ZEN-14-Glc and release of ZEN [39]. 

Our results with ZEN-14-Glc and ZEN-16-Glc hint at degradation of the glucosides in the digestive tract of piglets. Already in 1990, Gareis et al. [40] had shown that ZEN-14-Glc was completely metabolized to ZEN and α-ZEL during digestion in the pig. In contrast to our study, feed contaminated with ZEN-14-Glc was administered only to one pig (positive control) and another pig received a non-contaminated diet (negative control). In addition, the urine samples of the positive control animal were enzymatically hydrolyzed with β-glucosidase, which might have resulted in cleavage of ZEN-14-Glc. In the feces and urine samples of the negative control animal, no mycotoxins could be detected. In excreta samples of the positive control pig, ZEN and α-ZEL could be found. The limited number of pigs used in this study does not permit general conclusions on the metabolism of ZEN-14-Glc. 

In the study by Veršilovskis et al. [41] who exposed rats to an oral dose of ZEN-14-Glc, D3G and fully ^13^C-labeled ZEN and DON, the observation that ZEN was released from ZEN-14-Glc was supported. Unlabeled ZEN was detected in the stomach, confirming that the glucoside was partly hydrolyzed in the upper part of the GI tract. Additionally, ZEN-14-GlcA was identified in the intestines. Much lower levels of ZEN-14-Glc were determined in the small intestine. In the colon, greater amounts were determined than in the small intestine, which suggests that ZEN-14-Glc was not fully hydrolyzed. As we could not detect any glucosides in urine or feces of pigs dosed with ZEN glucosides, the fate of the modified toxins might differ between pigs and rats. In contrast to our study, 71% of ZEN equivalents and 74% of ^13^C-labeled ZEN was recovered in rats. The low recovery in our study might be due to investigation of another animal species with a different GI tract in which extensive hydrolysis by intestinal bacteria takes place. 

Since ZEN-16-Glc was only recently discovered as a masked mycotoxin [14], there is a lack of in vivo studies. The in vitro incubation of intestinal bacteria from human fecal slurry—which was collected from six donors—with ZEN-16-Glc showed that this analyte, too, was nearly completely converted to ZEN within 30 min. After two hours, no residue of ZEN-16-Glc was found in the fecal slurry [14]. The lower biological recovery of administered ZEN-16-Glc compared to ZEN-14-Glc might be explained by more extensive conversion of ZEN-16-Glc by intestinal bacteria. 

## 3. Conclusions

To sum up, the plant and fungal ZEN metabolites ZEN-14-Glc, ZEN-16-Glc, and ZEN-14-S were readily hydrolyzed to ZEN and converted to other still-unknown metabolites in the GI tract of pigs. As suggested in previous studies, the gut microbiota plays an important role and should be considered for further studies of xenobiotic bioactivity in animals and humans. In our work, the total amounts of ZEN-14-GlcA, ZEN and α-ZEL excreted into urine 0–48 h after administration of ZEN and its conjugates were greater for ZEN (26% ± 10%) than for its plant and fungal metabolites (between 13% ± 7% and 19% ± 11%). Inversely, ZEN glucoside metabolites were recovered to greater extent in feces, indicating reduced bioavailability of ZEN originating from ZEN conjugates. Despite slightly reduced toxicity compared to ZEN, the investigated modified forms of ZEN contribute to the total ZEN burden of an individual. Hence, we suggest considering the sum of ZEN and its naturally-occurring metabolites ZEN-14-Glc, ZEN-16-Glc, and ZEN-14-S as a group guidance (or maximum) value. For setting these values, further animal studies with chronic exposure to naturally-contaminated feed are required. 

## 4. Materials and Methods

### 4.1. Chemicals and Standard Solutions

Methanol (MeOH) and acetonitrile (ACN) (both LC grade) were purchased from VWR International GmbH (Vienna, Austria). Glacial acetic acid (LC-MS grade) and ethanol were obtained from Sigma-Aldrich (Vienna, Austria) and from Carl Roth GmbH and Co. KG (Karlsruhe, Germany), respectively. Water was purified with a Purelab Ultra system (ELGA LabWater, Celle, Germany).

For method validation, reference standard solutions of ZEN (100.8 µg/mL in ACN), α-ZEL (10.4 µg/mL in ACN), and β-ZEL (9.9 µg/mL in ACN) from Romer Labs (Tulln, Austria) were used. Solid ZEN-14-S was also obtained from Romer Labs. ZEN was previously purified from 24-day-old rice cultures inoculated with *Fusarium graminearum* (NRRL 66037) as described in Michlmayr et al. [42]. ZEN-glucosides were obtained by enzymatic conversion of ZEN by a barley UDP-glucosyltransferase (*Hv*UGT14077) and purified by preparative HPLC [42]. Lyophilized compounds were dissolved in ethanol for use as analytical standards and the stock solutions containing between 991 (ZEN-14-S) and 1274 (ZEN-14-Glc) µg/mL of the analytes were stored at −20 °C. For spiking experiments further dilutions were prepared in MeOH/water (20/80, *v/v*). ZEN- and ZEL-glucuronides were synthesized as described in Mikula et al. [34,43].

### 4.2. Animals and Study Design

Four castrated male weaning piglets (sow: Landrace × Large White, boar: Pietrain; 33–35 days old, 7.9 ± 0.6 kg) were obtained from a local producer in Lower Austria. Animals were housed separately in metabolic cages in total for 22 days and were allowed to acclimatize for five days before the start of the experiment. They had free access to water and feed was provided twice per day (semi ad libitum). The diet was analyzed for its concentration of ZEN and naturally occurring metabolites thereof.

Using a repeated measures design, the piglets (*n* = 4) received water (negative control), ZEN (10 µg/kg b.w.; positive control) and the equimolar dose of ZEN-14-S (12.5 µg/kg b.w.), ZEN-16-Glc (15.1 µg/kg b.w.) and ZEN-14-Glc (15.1 µg/kg b.w.) per gavage (orogastric application) on day 1, 3, 7, 11, and 15 of the experiment, respectively. From the stock solutions containing between 991 (ZEN-14-S) and 1274 (ZEN-14-Glc) µg/mL of the analytes the adequate volumes for the animal experiment were removed and diluted with water for the oral application to obtain 5 µg/mL ZEN and the equimolar concentration of the other analytes in the aqueous stock solutions. Using polyvinyl chloride catheters (diameter 4.7 mm, Ch 14, mpö pfm GesmbH (Klagenfurt, Austria), length was adapted to the size of the esophagus of the piglets), volumes between 16 and 30 mL of the aqueous stock solutions were orally administered to the piglets, considering the piglets’ weight. After the toxin application, 10 mL of water was used to flush the catheter. Urine and feces of each of the piglets were quantitatively collected for the periods 0–24 h and 24–48 h after dosing. Urine was measured volumetrically on site and all excreta samples were stored at −20 °C until lyophilization (feces) or sample preparation and analysis (urine). The general condition of the animals was observed daily during the experiment.

The animal experiment was approved by the institutional ethics committee and the national authority (LF1-TVG-39/014-2014, decision of 8 September 2014) according to § 26 of Animal Experiments Act, Tierversuchsgesetz 2012—TVG 2012 [44].

### 4.3. Sample Preparation

Urine samples were diluted 1:5000 with water and the creatinine content was determined by LC-MS/MS as described earlier [45]. For determination of ZEN, its plant and fungal metabolites and their metabolites in pigs, urine samples were diluted to 0.2 mM creatinine with water and centrifuged prior to HPLC-MS/MS analysis.

Five-hundred milligram aliquots of freeze-dried and thoroughly homogenized feces samples were extracted three times (90/30/30 min) with 5 mL each of ACN/water (50/50, *v/v*) on a GFL rotary shaker (type 3017, Burgwedel, Germany). After each extraction step, samples were clarified by centrifugation (10 min, 12,500 rpm, GS-15 centrifuge, Beckman Coulter, Indianapolis, IN, USA). Aliquots of the pooled supernatants were centrifuged and measured by HPLC-MS/MS. 

### 4.4. HPLC-MS/MS Parameters

Analysis of the feces and urine samples was performed on a 1290 Infinity series UHPLC system (Agilent Technologies, Waldbronn, Germany) coupled to a 6500 QTrap mass spectrometer equipped with an IonDrive TurboV source (Sciex, Foster City, CA, USA). Analyst software version 1.6.2 (Sciex, Foster City, CA, USA) was used for instrument control and data analysis. Chromatographic separation was carried out on a Kinetex Biphenyl column (150 mm × 3 mm, 2.6 µm, Phenomenex, Aschaffenburg, Germany). Eluent A consisted of water/acetic acid (99.9/0.1, *v/v*), eluent B was composed of ACN/acetic acid (99.9/0.1, *v/v*). After an initial period of 0.5 min at 15% B, the proportion of B was linearly increased to 60% at 13.5 min. At 14 min, 100% was reached which was held until 16.9 min. Afterwards, the column was re-equilibrated at 15% B for 3 min, resulting in a total run time of 20 min. The flow rate was 400 µL/min, the column temperature was 30 °C and the injection volume was 2 µL. Mass spectrometric detection was performed in negative electrospray ionization mode and selected reaction monitoring (SRM) was applied as scan type. The source settings were as follows: source temperature 400 °C, ion spray voltage −4500 V, curtain gas 35 psi (ca. 240 kPa), ion source gas 1 at 55 psi (ca. 379 kPa) and gas 2 at 65 psi (ca. 448 kPa), collision gas (nitrogen) high. SRM transitions were optimized by syringe pump infusion of analyte solutions and software controlled parameter optimization. Optimized parameters are provided in Table 5. Due to matrix interferences in real samples, the transition with lower intensity of α- and β-ZEL was used as quantifier.

### 4.5. Method Validation

The validation of the sample preparation and analysis methods for feces and urine included determination of the apparent recovery (R_A_), matrix effects (=signal suppression/enhancement, SSE), recovery of the extraction step (R_E_), the repeatability (RSD_r_), linearity, and the limits of detection (LODs) and quantification (LOQs). For determination of the R_A_ in feces, 500 mg aliquots of lyophilized and thoroughly homogenized blank feces of piglets were spiked with ZEN, α-ZEL, β-ZEL, ZEN-14-Glc, ZEN-16-Glc, and ZEN-14-S at eight different spiking levels, ranging from 3 to 900 ng/g of the individual compounds in lyophilized feces. By adding 50 µL of standard mixtures containing analytes at concentrations between 0.03 and 9 µg/mL, samples were spiked in triplicate before extraction. After addition of the analytes, the extraction of the spiked and three non-spiked feces samples was carried out as described above. Additionally, at those levels corresponding to the concentrations of analytes in feces samples after extraction, neat solvent and matrix matched calibration functions were prepared. By comparison of slopes of standard addition before extraction, matrix matched and neat solvent calibration functions, R_A_s, R_E_s, and SSEs were calculated [46]. For determination of the R_A_ in urine, 950 µL of pooled blank urine (standardized to 0.2 mM creatinine) was spiked in triplicate with 50 µL of standard mixtures containing ZEN, α-ZEL, β-ZEL, ZEN-14-Glc, ZEN-16-Glc, ZEN-14-S, α-ZEL-GlcA, β-ZEL-GlcA and ZEN-14-GlcA at 10 different spiking levels, corresponding to a linear working range between 0.1 and 100 ng/mL in the measurement solution for urine. A neat solvent calibration function was prepared at the same spiking levels. Recoveries were calculated according to Sulyok et al. [46]. The limits of detection and quantitation were determined in neat standard solutions and matrix matched standard solutions from the spiking levels closest to a signal-to-noise ratio (S/N) of 3:1 (=LOD) and 10:1 (=LOQ). The LODs and LOQs in freeze-dried feces were calculated by multiplication with the dilution factor. By triplicate work-up and measurement of feces and urine samples spiked at eight and 10 concentration levels, respectively, the repeatabilities (RSD_r_s) were evaluated for every concentration level, and averaged afterwards. 

### 4.6. Analysis of Samples and Data Evaluation

Urine and feces samples were analyzed in duplicate and the average of the duplicates was calculated. If the duplicates varied more than 20%, the samples were worked up again and re-measured. On the basis of pure solvent calibration functions (peak area versus analyte concentration, 1/x weighted calibration) established between 0.1 and 30 ng/mL using Analyst software, the quantitation of analytes in feces sample extracts was performed. The calculated concentrations were corrected by the R_A_ average values and multiplied by the dilution factor. The quantitation of ZEN and its metabolites in urine was carried out on the basis of pure solvent calibration functions by plotting peak areas against analyte concentrations established between 0.1 ng/mL and 100 ng/mL. The calculated concentrations were again corrected by the R_A_ average values and the dilution factor. If feces/urine samples showed signal-to-noise ratios between 3:1 and 10:1, LOQ/2 was used for further calculations. By multiplying the total amount of lyophilized feces samples or the total volume of urine samples (which were collected from each animal separately 24 h and 48 h after toxin application) with the corrected calculated concentrations, the total amounts of excreted ZEN and its metabolites were calculated. The total biological recovery considering the actual administered amount of the toxin was calculated by dividing the molar amount of excreted ZEN and its metabolites by the molar amount of applied toxin.

## Figures and Tables

**Table 1:** Method validation parameters for pig feces.

Analyte	R_A_^a^ ± RSD ^e^ (%)	SSE ^b^ ± RSD ^e^ (%)	R_E_^c^ ± RSD ^e^ (%)	LOD ^d^ in Solution (ng/mL)	LOQ ^d^ in Solution (ng/mL)
α-ZEL ^f^	49 ± 14	47 ± 15	103 ± 12	0.08	0.28
β-ZEL ^g^	104 ± 5	75 ± 16	139 ± 14	0.30	1.0
ZEN ^h^	85 ± 9	77 ± 9	113 ± 11	0.10	0.33
ZEN-14-Glc ^i^	75 ± 3	66 ± 4	114 ± 5	0.06	0.19
ZEN-16-Glc ^j^	86 ± 5	75 ± 7	115 ± 6	0.73	2.4
ZEN-14-S ^k^	124 ± 5	104 ± 5	119 ± 4	0.21	0.70

^a^ Apparent recovery; ^b^ Signal suppression/enhancement; ^c^ Extraction recovery; ^d^ Limits of detection and quantification in spiked blank feces extract; ^e^ Relative standard deviation; ^f^ α-zearalenol; ^g^ β-zearalenol; ^h^ zearalenone; ^i^ zearalenone-14-*O*-*β*-glucoside; ^j^ zearalenone-16-*O*-*β*-glucoside; ^k^ zearalenone-14-sulfate.

**Table 2:** Method validation parameters for pig urine.

Analyte	R_A_^a^ ± RSD ^c^ (%)	LOD ^b^ in Solution (ng/mL)	LOQ ^b^ in Solution (ng/mL)
α-ZEL ^d^	89 ± 8	0.11	0.38
β-ZEL ^e^	84 ± 9	0.16	0.54
ZEN ^f^	95 ± 9	0.15	0.49
ZEN-14-Glc ^g^	88 ± 9	0.07	0.23
ZEN-16-Glc ^h^	96 ± 9	0.18	0.60
ZEN-14-S ^i^	98 ± 10	0.02	0.08
α-ZEL-GlcA ^j^	113 ± 6	0.73	2.4
β-ZEL-GlcA ^k^	110 ± 7	0.42	1.4
ZEN-14-GlcA ^l^	96 ± 9	0.30	1.0

^a^ Apparent recovery; ^b^ Limits of detection and quantification in spiked diluted blank urine; ^c^ Relative standard deviation; ^d^ α-zearalenol; ^e^ β-zearalenol; ^f^ zearalenone; ^g^ zearalenone-14-*O*-*β*-glucoside; ^h^ zearalenone-16-*O*-*β*-glucoside; ^i^ zearalenone-14-sulfate; ^j^ α-zearalenol-14-glucuronide; ^k^ β-zearalenol-14-glucuronide; ^l^ zearalenone-14-glucuronide.

**Table 3:** Excretion of zearalenone (ZEN) and its metabolites in feces and urine of piglets. Values are average values (*n* = 4) per treatment and time period. Individual urine and feces samples were analyzed in duplicate and the average values of the duplicates were used for calculation of average values within one treatment.

Average Amounts Excreted (nmol ± std. dev. ^a^) ^b^						
Treatment	Matrix	Time Period	ZEN ^c^	α-ZEL ^d^	ZEN-14-GlcA ^e^	Total ^f^
ZEN	Feces	0–24 h	7	4 (*n* = 1)	n.d. ^g^	8
		24–48 h	24	13	n.d.	37
		**0–48 h**	**31 ± 9**	**14 ± 6**	**n.d.**	**45 ± 14**
	Urine	0–24 h	20 (*n* = 3)	n.d.	62 (*n* = 3)	62
		24–48 h	13 (*n* = 3)	n.d.	13 (*n* = 2)	16
		**0–48 h**	**33 ± 12 (*n* = 3)**	**n.d.**	**53 ± 44**	**78 ± 31**
ZEN-14-S ^h^	Urine	0–24 h	44	8 (*n* = 1)	44 (*n* = 1)	57
		24–48 h	11	n.d.	n.d.	11
		**0–48 h**	**55 ± 19**	**8 (*n* = 1)**	**44 (*n* = 1)**	**68 ± 21**
ZEN-14-Glc ^i^	Feces	0–24 h	40	21 (*n* = 2)	n.d.	50
		24–48 h	48	29	n.d.	77
		**0–48 h**	**88 ± 21**	**40 ± 19**	**n.d.**	**127 ± 32**
	Urine	0–24 h	52	17 (*n* = 1)	46 (*n* = 2)	80
		24–48 h	n.d.	n.d.	21 (*n* = 1)	21 (*n* = 1)
		**0–48 h**	**52 ± 15**	**17 (*n* = 1)**	**57 ± 45 (*n* = 2)**	**85 ± 46**
ZEN–16–Glc ^j^	Feces	0–24 h	25	12 (*n* = 2)	n.d.	31
		24–48 h	36	16	n.d.	52
		**0–48 h**	**62 ± 21**	**22 ± 7**	**n.d.**	**84 ± 23**
	Urine	0–24 h	32	19 (*n* = 2)	18 (*n* = 1)	46
		24–48 h	9 (*n* = 2)	n.d.	n.d.	9 (*n* = 2)
		**0–48 h**	**36 ± 19**	**19 ± 3 (*n* = 2)**	**18 (*n* = 1)**	**51 ± 28**

^a^ As the excreted amounts varied widely in the individual time periods, the standard deviation was only calculated for the total time period of excreta collection (0–48 h); ^b^ *n* = 4 unless stated otherwise. Values < LOD were not considered in the calculation of average values. Traces (values between LOD and LOQ) were included as LOQ/2; ^c^ zearalenone; ^d^ α-zearalenol; ^e^ zearalenone-14-glucuronide; ^f^ First, the total amount of metabolites excreted into urine or feces was calculated separately for each animal and time period. Then, the average for each treatment, matrix and time period was calculated (*n* = 4 unless stated otherwise); ^g^ n.d.: not detected; ^h^ zearalenone-14-sulfate; ^I^ zearalenone-14-*O*-*β*-glucoside; ^j^ zearalenone-16-*O*-*β*-glucoside.

**Table 4:** Biological recoveries in feces and urine of piglets (*n* = 4).

Biological Recovery ± std. dev. (%) ^a,b^					
Matrix	Time Period	ZEN ^c^	ZEN-14-S ^d^	ZEN-14-Glc ^e^	ZEN-16-Glc ^f^
Feces	0–24 h	3 ± 2	n.d. ^g^	12 ± 13	9 ± 11
	24–48 h	12 ± 4	n.d.	17 ± 5	13 ± 7
	**0–48 h**	**14 ± 3**	**n.d.**	**29 ± 8**	**22 ± 7**
Urine	0–24 h	20 ± 11	16 ± 5	18 ± 9	11 ± 6
	24–48 h	6 ± 2	3 ± <1	5 (*n* = 1)	2 ± <1 (*n* = 2)
	**0–48 h**	**26 ± 10**	**19 ± 5**	**19 ± 11**	**13 ± 7**
Total	0–24 h	22 ± 10	16 ± 5	30 ± 9	20 ± 5
	24–48 h	18 ± 3	3 ± <1	18 ± 7	14 ± 7
	**0–48 h**	**40 ± 8**	**19 ± 5**	**48 ± 7**	**34 ± 3**

^a^ *n* = 4 unless stated otherwise; ^b^ First, the biological recovery was calculated separately for each animal and time period. Then, the average for each treatment, matrix and time period was calculated; ^c^ zearalenone; ^d^ zearalenone-14-sulfate; ^e^ zearalenone-14-*O*-*β*-glucoside; ^f^ zearalenone-16-*O*-*β*-glucoside; ^g^ n.d.: not detected.

**Table 5:** Optimized selected reaction monitoring (SRM) parameters.

Analyte	Retention Time (min)	[M − H]^−^ (*m/z*)	DP ^c^ (V)	Product Ions (*m/z*) ^a^	CE ^d^ (eV)	Relative Intensity ^b^
ZEN ^e^	13.20	317.1	−120	131.0/175.0	−42/−34	0.70
α-ZEL ^f^	11.22	319.1	−125	160.0/275.1	−30/−42	2.7
β-ZEL ^g^	10.12	319.1	−125	160.0/275.1	−30/−42	2.6
ZEN-14-Glc ^h^	8.80	479.1	−125	317.0/175.0	−22/−54	0.10
ZEN-16-Glc ^i^	7.35	479.2	−140	149.0/160.8	−54/−54	0.49
ZEN-14-S ^j^	8.89	397.0	−115	317.0/131.0	−34/−58	0.08
ZEN-14-GlcA ^k^	8.50	493.1	−115	317.0/175.0	−36/−26	0.36
α-ZEL-GlcA ^l^	7.23	495.1	−110	319.0/112.8	−38/−28	0.31
β-ZEL-GlcA ^m^	6.13	495.1	−110	319.0/112.8	−38/−28	0.30

^a^ Product ions: quant/qual; ^b^ Relative intensity: qual/quant; ^c^ Declustering Potential; ^d^ Collision Energy; ^e^ zearalenone; ^f^ α-zearalenol; ^g^ β-zearalenol; ^h^ zearalenone-14-*O*-*β*-glucoside; ^i^ zearalenone-16-*O*-*β*-glucoside; ^j^ zearalenone-14-sulfate; ^k^ zearalenone-14-glucuronide; ^l^ α-zearalenol-glucuronide; ^m^ β-zearalenol-glucuronide.
